# Routine surveillance for the identification of drug resistant Tuberculosis in Tanzania: A cross-sectional study of stakeholders’ perceptions

**DOI:** 10.1371/journal.pone.0212421

**Published:** 2019-02-22

**Authors:** Basra Esmail Doulla, Stephen Bertel Squire, Eleanor MacPherson, Esther Stanslaus Ngadaya, Beatrice Kemilembe Mutayoba, Ivor Langley

**Affiliations:** 1 Ministry of Health, Community Development, Gender, Elderly and Children; National Tuberculosis and Leprosy Programme, Dar es Salaam, Tanzania; 2 Liverpool School of Tropical Medicine, Centre for Applied Health Research and Delivery, Liverpool, United Kingdom; 3 National Institutes for Medical Research, Muhimbili Medical Research Centre, Dar es Salaam, Tanzania; Vietnam NTP, VIETNAM

## Abstract

**Background:**

Routine surveillance is required to monitor the performance of tuberculosis diagnostic programme and is essential for the rapid detection of drug resistance. The main objective of this study was to explore the effectiveness and stakeholder perception of the current routine surveillance system for previously treated tuberculosis cases in Tanzania with a view to identify interventions to improve and accelerate positive patient outcomes.

**Methods:**

A study using quantitative and qualitative methods of data collection including in-depth interviews and focus group discussions with health care service providers was conducted in four regions. Quantitative data were extracted from the routine databases to assess performance.

**Results:**

Quantitative findings from 2011 to 2013 showed 2,750 specimens from previously treated TB cases were received at the reference laboratory. The number increased year on year, but even in the most recent year was only 61% of that expected. The median and interquartile range of turnaround time in days from specimen reception to results reported for smear microscopy, culture and drug susceptibility testing were 1(1, 1), 61(43, 71) and 129(72, 170) respectively. Contaminated specimens were reported in 3.6% of cases. The qualitative analysis showed the system of sending specimens using postal services was seen to be efficient by participants. However, there were many challenges and significant delays in specimens reaching the reference laboratory associated with lack of funds to transfer specimens, weak form completion, inadequate training and poor supervision. These all adversely affected the implementation of the routine surveillance system.

**Conclusions:**

Many issues limit the effectiveness of the routine surveillance system in Tanzania. Priority areas for strengthening are; specimen transportation, supervision and availability of commodities. A pilot study of a revised routine surveillance system that takes into account the observations from this study alongside improved access to drug susceptibility testing using Xpert MTB/RIF should be considered.

## Introduction

Tuberculosis (TB) remains a major global health problem alongside Human Immunodeficiency Virus (HIV) /Acquired Immune Deficiency Syndrome (AIDS) as a leading cause of death [1]. Global TB control efforts have made progress in the past decade, however despite this in 2016 the World Health Organization estimated there were 10.4 million incident cases of TB and 1.4. million deaths from TB. 11% of the incident cases were people living with HIV. WHO estimates 4 million were cases were undiagnosed. [1]. Drug-resistant TB is a threat across the globe with estimated incidence of 480,000 Multidrug Resistant TB (MDR-TB) cases, of which 9.5% were Extensively Drug Resistant TB (XDR-TB). [1]. In the same year only 30% of the 3.4 million new bacteriologically confirmed and previously treated TB cases notified, were reported to have been tested for resistance to Rifampicin (one of the key drugs used to treat TB in the standard TB regimen).[1]. The development of XDR-TB has added a new dimension to the threat of the TB epidemic and highlights the inadequate resources available in many settings. [2]. The need for reliable drug-susceptibility testing and an appropriate shorter course MDR-TB treatment regimen has increased with the growth in incidence in many parts of the world. [3]. Application of molecular methods during the last decade has greatly changed the understanding of drug resistance in TB. [4]. In 2010 the World Health Organisation (WHO) endorsed the use of Xpert MTB/RIF assay in low and middle-income countries. [5]. Tanzania is among the 30 countries with the highest TB burden worldwide according to the WHO. [1]. In Tanzania 63,151 TB cases were notified in 2015 of these 60,563 were new cases and 1,580 were previously treated TB cases. [1]. The MDR-TB rate estimation from the 2006 Drug Resistance Survey (DRS) was 1.1% and 3.1% in new and previously treated TB cases respectively. [6]. The WHO recent estimates are 1.3% for new and 4.7% of previously treated cases. [7]. The WHO recommends using more rapid and sensitive diagnostic methods for drug resistance testing. [8]. At the time of the study Acid Fast Bacilli smear microscopy was the main TB diagnostic tool in the country and there were 735 TB centres. There were two Zonal TB culture laboratories and only one standalone Central TB Reference Laboratory with capacity to routinely perform culture and drug susceptibility testing for patient monitoring and drug resistant identification. [9]. The National TB and Leprosy Programme introduced Xpert MTB/RIF in 2012 at two regional hospitals for evaluation of the technique. In 2013 Xpert MTB/RIF was installed in thirteen health facilities but without standard guidelines or an algorithm from the TB Programme.

The main purpose of the routine surveillance system in Tanzania is to monitor the TB Programme at the district, regional and national levels. This includes identifying individuals with drug resistance and informing the districts, so they can be put the patient on appropriate treatment as early as possible. The current Routine Surveillance System requires, morning specimens from 25% of new cases and 100% of previously treated cases to be routinely collected at the peripheral level and, sent to the Central TB Reference Laboratory in Dar es Salaam from the districts and regions. Transportation uses public transport or Expedited Mail Services (EMS). [9]. This study focuses on the previously treated cases because that is where most MDR-TB cases are found. [6,10], although lessons learnt for previously treated cases are also likely to be relevant to new TB cases. In addition, failure to appropriately track these cases could lead to emergence of XDR-TB in the country, so understanding whether (and if not–why not?) drug susceptibility testing was taking place for all previously treated cases was considered critical. Effective specimen shipment arrangements are essential to ensuring all specimens are received and tested at the reference laboratory. In addition, transportation can effect specimen viability, speed of results feedback and therefore the quality of patient management.

Previously treated TB cases submit specimens throughout the year to the Central Reference Laboratory. Results are sent back to Regional TB Coordinators who then distribute them to the requesting site.

The study noted that, since the TB Programme’s inception in 1977 there has never been any systematic evaluation of the routine surveillance system. A study is therefore much needed to identify the system’s pitfalls and develop interventions that could lead to improved identification of drug resistance and to avert the spread of drug resistant TB.

### Objectives of the study

The main objective of this study was to explore the effectiveness and stakeholder perception of the current routine surveillance system for previously treated tuberculosis cases in Tanzania with a view to identifying interventions to improve and accelerate positive patient outcomes.

## Methods

### Study sites

The quantitative part of the study reviewed the country routine TB laboratory data collected electronically from TB laboratory registers at the Central TB Reference Laboratory from January 2011 to December 2013. The dataset contained information on all the specimens received at the Central TB Reference Laboratory from across the country. [9].

The information from the quantative part of the study fed into the design of the qualitative part of the study which covered four regions purposely selected with the highest levels of notified TB cases in Tanzania in the year 2012. [11]. The regions were Dar es Salaam (21.9% of all cases in Tanzania), Arusha (4.9%), Morogoro (5.1%) and Shinyanga (6.4%). [11].

#### Study design

A cross-sectional study that employed both quantitative and qualitative data collection was conducted. The quantitative approach was used to analyse routine data collected from January 2011 to December 2013 to understand the volumes, timings and results from the current routine surveillance system (RSS). A qualitative approach was used to seek to understand from stakeholders working in the programme and involved in the RSS how they perceived the current RSS in order to understand its strengths, weaknesses and how it might be improved from their perspective.

The quantitative data collected included information on completion of request forms, transit times, turnaround time, culture positivity and contamination rates. The transit time is the time between sputum collection at the peripheral site and the specimen receipt at the Central TB Reference Laboratory. According to the National TB and Leprosy Program the anticipated transit time should be a maximum of 7 days. [9]. Turnaround time is defined as the time between a specimen arriving at the Central TB Reference Laboratory and results being reported back to the requesting facility. [12]. The expected turnaround time for smear microscopy is 1 day, culture is 56 days, and drug susceptibility testing is 42 days. [8].

The qualitative data collection focused on stakeholders purposely sampled including members of the health system staff at the National TB and Leprosy Programme, the Central TB Reference Laboratory, and health care workers at regional/district level. Implementing partners supporting the National TB and Leprosy Programme and Ministry of Health Officials were also included. Qualitative methods used were in-depth interviews (IDI) and Focus Group Discussions (FGD). These methods gave maximum opportunity for different perspectives from diverse groups to be gathered and ensured the findings would be broad and representative. Oral and written explanations of the aims and expected benefit of the study were provided. All participants willingly participated and were made aware of the tape-recording of interviews. The research assistants emphasised the importance of confidentiality throughout the study.[13] The Principal Investigator provided training to the research team on how to pose open ended questions and observe nonverbal communication. All interviews were transcribed from tape recordings and transcriptions were imported to NVIVO 10 software for analysis. Reports were translated from Swahili into English. To ensure good quality of the transcriptions, the principal investigator checked 5% of the transcripts. The interviews were read and re-read to find the key messages from the frequency of similar responses. Key themes and sub-themes were developed from these messages.

#### Sample size and sampling procedures

A total of six FGDs were conducted involving 45 participants (25 males and 20 females). 10 participants were interviewed using the IDI (6 males and 4 females). The selection of participants was done purposively for Regional TB Coordinator as there is only one per region. [14]. District TB Coordinator, TB nurse, courier person, program staff and TB laboratory person at all levels were conveniently selected.[15]. The Central TB Reference Laboratory had twenty staff, therefore the sampling strategy of selecting study participants for a small population (i.e. less than fifty) was adopted.[16]. At each selected study site, members were requested to take part in the FGD, there were at least six to eight participants in each FGD per region. FGDs were guided by two research assistants, one leading the discussion and the other observing and writing down main points for each theme. IDIs were conducted with ministry officials, regional/district administrators and implementing partners supporting the TB Programme.

#### Data collection

The two local research assistants (one female and one male) involved in both IDIs and FGDs benefited from three weeks training. Each research assistant alternated between interviewing the participants and taking notes. The interviews lasted 1–1.5 hours. The data were collected on paper interview forms, Table 1 for FGD and Table 2 for IDI.

**Table 1:** Topic guide for focus group discussion.

**S/N**	TB routine surveillance system	Specimen Transportation	Central TB Reference Laboratory	Relationship to the workload	Knowledge on TB	Concluding the discussion
1	How are TB health care workers involved in the routine surveillance system?	Does the clinic transport specimens to another laboratory?	What is the perception of the health care workers on the TB reference laboratory?	Where do your patients come from?	How do health care workers become aware of new TB diagnostic strategies?	Is there anything else you think would be useful for the programme to know?
2	Probe; what is their roles in RSS, what do they think of the current method, what is their perception on the TB Programme and the reference laboratory?).	Who does the specimen transportation? (Probe; how is it transported?)	What do they think could be done better or could be sustained?	probe; (how many patients per day per clinic, if heavy clinic, why, issues around health education and who does the TB screening)	(Probe; from the programme, training, internet, collaboration with the TB programme, how is it disseminated)	Do you have any questions about any aspect of this interview?
3	Describe how routine surveillance system is implemented in your health facility?	Probe means of specimen transportation	(Probe: Issues around TB networking, results turnaround time and the performance).	What perceptions do the health workers have towards patient management?	What do health care workers know about TB transmission?	
4	Probe; if health care workers know about the routine surveillance system and what type of specimens need to be sent, algorithm and safety)			(Probe; compared to the number of staff, skills, motivation)	Probing on ways it is transmitted, ways it is prevented, explore questions relating to TB diagnosis)	
5	What do health care workers think are the challenges in implementing the routine surveillance system?				What knowledge do the health care workers have on RSS?	
6	Probe issues related to: causes that contribute to TB diagnosis delays, is it the patients or the programme, feedback of the results, specimen handling and support from the TB Programme).				Probe; how it works, collaboration with the reference lab, specimen to be transported, frequency)	
7	What could be done differently to make it better, what could be beneficial to the community?					

Key: TB- Tuberculosis; RSS–Routine surveillance system

**Table 2:** Topic guide for In-depth interview.

S/N	Heath workers Views and Roles	Health workers perception	Knowledge on RSS	Perceptron on the Network	Closing
1	What are your views about the TB control Programme performance?	Ask for their perspective; is there anything, which would prevent them from changing the current services provided or the system?	What do you know about TB routine surveillance system?	Probe what is their perception of the TB networking?	Is there anything else you think would be useful for programme to know? Do you have any questions about any aspect of this interview?
2	What do you regard as the successes of routine surveillance system?	What have been some of the challenges for you as an individual?	What recommendation would you make on RSS?	Any other comments please?	
3	How do you view your own role in the TB programme? (Probe)				
4	How do you see your role in supporting TB programme policies implementation?				

Key: TB- Tuberculosis; RSS–Routine surveillance system

To maintain participant’s confidentiality, participant’s names were not captured during the entire study, instead study numbers; group numbers; and district/region codes were used. As the principal investigator is currently responsible for overseeing the entire TB laboratory services in the country it was felt that using a research assistant would ensure participants were comfortable in answering the questions honestly. The interviews utilised key themes such as: Effectiveness and perception of the TB program; routine surveillance system implementation in Tanzania; Diagnostic method and Centralization of TB Laboratory services; Communication between the Central TB Reference Laboratory and the districts.

#### Data analysis

**Quantitative data:** Quantitative data were extracted from the routine database at the Central TB Reference Laboratory and TB laboratory registers covering the three-year period. The collected data were cleaned and analysed using Statistical Package for Social Science (SPSS) version 23. Frequency tables were generated to explore outliers and abnormal values which were then rectified using the available source documents. The transit time (time from specimen collection from the health facility to the time the specimen was received at the Central TB Reference Laboratory) and the turnaround time for TB tests performed at the laboratory were calculated. The proportion of the specimens tested within the recommended time [17,18] was also calculated and coded as 1, and those outside this time coded 0. Then descriptive analysis was performed on the proportional of specimens recorded as out of recommended time.

**Qualitative data:** Data from the IDI and FGD were analysed using the framework approach. [19]. Manual analysis began by preparing daily summaries after FGDs. The tape recorded FGDs were transcribed verbatim in Swahili by a transcriber. Each transcribed FGDs were reviewed for accuracy by replaying each recorded interview whilst reading and translating the transcripts. Thereafter, thematic analysis was carried out by assigning data into relevant themes in order to generate information on study objectives. All transcripts were imported and analysis using NVIVO 10 for the sixteen IDI and six FGDs. The research team developed a preliminary thematic framework which was used to code the transcripts. As new themes emerged from the transcribed data the framework was updated. The analysis came to an end once the theoretical saturation point had been reached. [13].

### Ethical clearance

Ethical clearance was granted by the Liverpool School and Tropical Medicine Research Ethical Committee in the United Kingdom and the National Health Research Ethical Review Committee from National Institute for Medical Research in Tanzania. The Principal investigator sought permission from the National TB and Leprosy Programme and Regional Administrative Authorities to conduct the study. Permission was granted by both establishments. Each participant gave a written consent prior to taking part in the study. Participants were made aware that, they are free to withdraw from the study at any time, without prejudice. Confidentiality was maintained throughout the study. They were also informed that, recorded interviews would be kept up to seven years with their permission. During the interview, simple structured open-ended questions were used through guidance notes and topic guides. In addition, for the quantitative part the principle investigator obtained permission from the National TB and Leprosy Programme to use the routine laboratory data from the Central TB Reference Laboratory.

## Results: Quantitative findings

### Distribution pattern of specimens

The quantitative part of the study analysed routine specimens received at the Central TB Reference Laboratory during the time under review (2011–2013). There were 16,423 specimens in total including both new and previously treated TB cases. Of these 2,750 (16.7%) were from previously treated TB cases which was the focus of this study. The majority of these specimens (61.7%) were received in 2013 and most were from males (60.2%). (Table 3). 1,495(54.4%) were from the young adult’ population aged between 25 and 44 years. HIV status was known in 1,962 (71.4%) and of these 1,293 (65.9%) were HIV negative. A high proportion of specimens 1,118(40.7%) were from the Eastern Zone (Mtwara, Lindi, Dar es Salaam). A total of 98(3.6%) were recorded as contaminated. (Table 3).

**Table 3:** Previously treated TB cases specimens’ received at the Central TB Reference Laboratory from 2011 to 2013.

Variable	2011; N = 80	%	2012; N = 973	%	2013; N = 1,697	%	Total	%
**Zone**								
Northern	37	46.3	186	19.1	630	37	853	31.0
Southern	3	3.8	115	11.8	94	5.5	212	7.7
Eastern	23	28.8	459	47.2	636	38	1,118	40.7
Lake/Central	17	21.3	210	21.6	315	19	542	19.7
Zone unknown	0	0	3	0.3	22	1.3	25	0.9
**Gender**								
Male	51	63.8	468	48.1	1,137	67	1,656	60.2
Female	29	36.3	319	32.8	550	32	898	32.7
Gender unknown	0	0	186	19.1	10	0.6	196	7.1
**Age group**								
<15	2	2.5	10	1	29	1.7	41	1.5
15–24	8	10	137	14.1	262	15	407	14.8
25–34	21	26.3	294	30.2	444	26	759	27.6
35–44	21	26.3	255	26.2	460	27	736	26.8
45–54	24	30	277	28.5	502	30	803	29.2
Age unknown	4	5	0	0	0	0	4	0.1
**HIV status**								
Positive	14	17.5	206	21.2	449	27	669	24.3
Negative	13	16.3	443	45.5	837	49	1,293	47.0
HIV Unknown	53	66.3	324	33.3	411	24	788	28.7
**TB type**								
Pulmonary TB negative	0	0	4	0.4	1	0.1	5	0.2
Pulmonary TB positive	67	83.8	957	98.4	1,685	99	2,709	98.5
Extra pulmonary TB	2	2.5	3	0.3	1	0.1	6	0.2
TB type not specified	11	13.8	9	0.9	10	0.6	30	1.1
**Smear result**								
Positive	36	45	645	66.3	993	59	1,674	60.9
Negative	44	55	328	33.7	704	42	1,076	39.1
**Culture result**								
Positive	34	42.5	392	40.3	755	45	1,181	42.9
Negative	45	56.3	532	54.7	879	52	1,451	52.8
Contamination	1	1.3	44	4.5	53	3.1	98	3.6
Result not documented	0	0	5	0.5	10	0.6	20	0.7

Keys: HIV- Human Immunodeficiency Virus; N- Number; TB- Tuberculosis.

Zones are Eastern (Mtwara, Lindi, Dar es Salaam); Northern (Arusha, Kilimanjaro, Tanga, Manyara); Southern (Iringa, Mbeya, Ruvuma, Katavi) Central (Dodoma, Morogoro, Singida) and Lake (Mwanza, Shinyanga, Tabora, Kagera, Kigoma, Geita)

### Relation between cases notified versus specimens received

**Fig 1:**
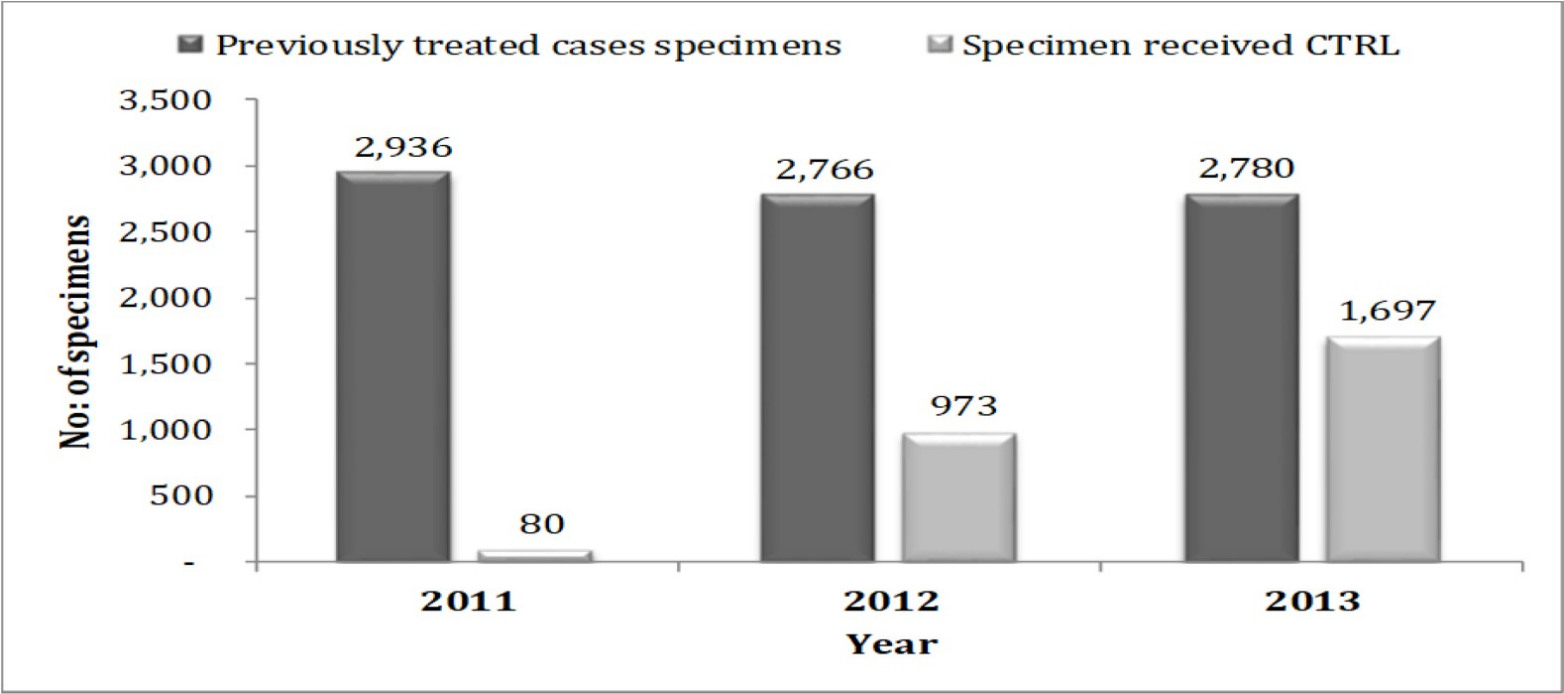
Previously treated cases notified versus specimen received at the Central TB Reference Laboratory– 2011 to 2013.

Analysis of the data showed that, out of 8,482 previously treated cases notified across the country for the three years, only 2,750 (32.4%) were received at the Central TB Reference Laboratory. A significant variation was recorded in 2011 where 2,936 cases were notified and only 80(2.7%) were received at the Central TB Reference Laboratory. The trend improved 2013 whereby 2,780 cases notified and 1,697 (61.0%) specimens received at the Central TB Reference Laboratory. (Fig 1).

Transit Time (Time between sputum collection and arrival at Central TB Reference Laboratory)

The study findings showed that, 2,608 (94.8%) of previously treated case specimens had documented transit times. The mean and median transit time was 8.1 and 6(IQR; 0–68) respectively with a full range of 0 to 100 days. The mean and median transit time was lower 6.1 and 3(IQR; 1–6.6) in 2011 compared to 2012 and 2013, though the different between the years was not statistically significant (P = 0.271). (Table 4).

**Table 4:** Transit time for previously treated cases from 2011 to 2013.

Year	Number	Mean transit time	Standard Deviation	Median transit time	p25	p75	F-value	p-value
2011	76	6.1	6.9	3	1	6.5	1.3	0.271
2012	911	8.4	12.2	6	4	9		
2013	1621	8.1	11.7	5	3	9		
Total	2,608	7.7	8.4	6	3	9		

Keys: p25- is the 25^th^ percentile (1^st^ quartile); p75- is the 75^th^ percentile (3^rd^ quartile).

Turnaround Time (Time between specimen arrival at Central Reference Laboratory and results feedback)

Findings presented from the total 2,750 previously treated TB cases specimens received: Specimens documented and processed within the recommended turnaround time were 1,024(82.3%) for microscopy; 146(42.4%) for culture and 11(2.8%) for drug susceptibility testing. (Table 5).

**Table 5:** Turnaround time for previously treated cases’ specimens from 2011 to 2013.

S/N	Status	Microscopy	Culture	Drug susceptibility testing
1	Within recommended time	1,024(82.3%)	1,146(42.4%)	11(2.8%)
2	Out of recommended time	220(17.7%)	1,557(57.6%)	381(97.2%)
	Total	1,244	2,703	392

The study findings showed that, from the previously treated TB case specimens analysed (2,750), only1,181(42.9%) were culture positive. From the positive cultures only 392 (33.2%) were set up for drug susceptibility testing and out of those 373(95.1%) obtained drug susceptibility testing results. Of those 269 (72.1%) were susceptible to all first line anti TB drugs. 72(19.3%) were MDR-TB, 27(7.2%) were mono-resistant and the remaining 5(1.3%) had other patterns of resistance. (Table 6).

**Table 6:** Drug susceptibility testing results profile from 2011–2013.

Year	2011	2012	2013	Total	% of Total
**Positive Culture**	34	392	755	1,181	100
DST Not Done	6	235	548	789	66.8
Total number set up for DST	28	157	207	392	33.2
Total number with DST results	28	151	194.0	373	31.6
Total number DST contaminated	0	6	13	19	1.6
**DST Patterns**					** % of Results**
Total Susceptible to all	16	123	130	269	72.1
Total MDR TB	9	16	47	72	19.3
Total Mono resistant	3	12	12	27	7.2
Total other resistant	0	0	5	5	1.3
**MDR TB**					
HRES	5	8	18	31	8.3
HRS	4	1	14	19	5.1
HR	1	7	14	22	5.9
**Mono resistant**					
H	1	5	2	8	2.1
S	1	1	3	5	1.3
R	1	6	7	14	3.8
**Other resistant**					
HS	0	0	4	4	1.1
ES	0	0	1	1	0.3

DST- drug susceptibility testing; MDR- multidrug resistant tuberculosis; TB- tuberculosis; HSRE; H-Isoniazid; R- Rifampicin; **S**-Streptomycin; E-Ethambutol.

### The qualitative findings

The key themes and sub-themes from the IDI and FGD’s are described below alongside example quotes from participants in the interviews and discussions.

### Effectiveness of the TB Programme

The findings explored how the routine surveillance system was understood by stakeholders. It was observed that, some aspects such as support to the districts and the use of the EMS for transportation of samples was helpful if sustained. Policy makers were said to be good in the FGD’s. However, some aspects such as staff demotivation, failure to follow good practices, delayed test results and a lack of rapid diagnostic technology were also noted.

*The following quote illustrates*: *“I think to be very sincere; the support they are giving us at peripheral level is tremendous*. *They are supportive and as far as the National TB and Leprosy Programme is concerned and very well organized*, *when we have a problem they will sort it out” (FGD –2014)”*.

### Routine surveillance system implementation in Tanzania

The routine surveillance system in Tanzania is a system where specimens are collected routinely and sent to the Central TB Reference Laboratory via EMS for additional testing (culture and drug susceptibility testing). The purpose is to identify drug resistant patients, so they can start treatment as early as possible. During FGD some males and two females suggested that the routine surveillance system needs improvement.

*The following quote illustrates*; *“Despite Routine Surveillance is working well*, *the way I see most of its targets have not reached*, *I suggest that it is better if the number of people responsible should circulate the information to all health care providers on how to do it for better understanding*.*”* ***(****FGD-2014)*.

*“Another challenges I have seen so far*, *is transportation is also quite a big challenge*. *Different areas*, *can be difficult to get specimens to reach the central level quick enough and the methods used to examine the specimens like the Lowenstein Jensen medium taking too long to get results and request forms not filled in properly”****(****FGD-2014)*.

Poor supervision was associated with staff demotivation.

*The following quote illustrates; “there is a need to strengthen supervision*, *make it more fruitful not just a vehicle visiting*. *It needs to be supportive*, *get there*, *stay with the staff*, *for them to recognize and listen to their problems and establish the hidden problems too then provide solution” (IDI– 2014)*.

*“If the supervision is done properly it will discover many things and resolve problems*.*” (FGD_2014)*

A male stakeholder said that, training was important in order to be up to date.

*As the quote below illustrate; “Education is not only done once*, *it is a continuous process*, *and people need to be taught regularly*, *provide people new techniques or to refresh them*.*” (IDI_2014)*.

Staff revealed that there are frequent stock outs of supplies.

*The following quote from a female member of staff illustrates*; *“Another challenge is sometimes there is lack or shortage of packaging materials and supplies*, *we used to receive these items from donor but their contract finished*, *this is now a problem*. *We need to know how to package specimens in layers to avoid leakages and specimen rejections at the Central TB Reference Laboratory” (IDI-2014)*

Due to failure to follow good practices, issues such as preparation of specimen packaging and form filling were among the things raised in interviews by two males.

*The following quote illustrates*; *“This is a long-standing problem laboratory request forms not filled in well*, *a lot of information is missing*. *We see forms coming with either one name or just initials and the rest of the information not filled in”*. *(FGD_2014)*.

Both male and female participants in FGD observed that in some districts, sputum specimens are sometimes delayed or interrupted this could be due to lack of funds for transportation.

*The following quotes illustrate*; *“In a parcel of specimens*, *you could find one specimen 15 days old and another 3 days old*, *something impossible*. *I think they get a specimen but they don’t send it on time*. *Instead they wait for them to be many before sending*. *(FGD_2014)*.

*We have a challenge on how to send those specimens*. *Who will take the specimens to the stations and who will pick the specimens up in Dar es Salaam and who will pay the costs for sending specimens*? *We had a partner but their contract ended*. *So*, *that is still hanging in the air waiting for someone else to kick in*.*” (IDI_ 2014)*.

A few male TB staff felt Dar es Salaam is less impacted compared to other regions.

*The following quotes illustrate*; **“***the transportation from Dar es Salaam Laboratory to the Central TB Reference Laboratory is not a problem*, *they can use bodaboda or EMS*. *Actually biggest problem is referring samples from peripheral laboratory to district laboratory where post services is not available;(IDI_2014)*.

During the FGD three males commented that there was no standard guideline for specimen referral or schedule for shipment.

*The following quotes illustrate*; *“First of all coordinators need to know the time specimen are to be sent to Dar es Salaam for culture*, *the awareness of the importance of those specimens should be there so that they would have enough containers and be able to truck the specimens as they are sent by* EMS.*” (FGD_2014)*.

### Diagnostic method and centralization of TB laboratory services

Some of the respondents asserted that, contamination problems lead to tests being repeated and there is a need to inform the clinicians about the duration and methods used.

*The following quote illustrates*; *“I think sometimes they are late as mentioned*, *there are many challenges*, *some examinations*, *whose specimen has spoilt(contaminated) that needs to be re-tested and that is when they are delayed and not intentionally” (IDI-2014)*.

Some felt that, there is a need to use rapid method for early diagnosis and patient management.

*The following quotes illustrates*; *“I would say the methods used to examine the specimens like the LJ medium method for culture and the drug susceptibility testing both take too long*. *I think it is a big challenge because we need to be able to get these results quicker*, *for instance*, *they could be examined by liquid culture and drug susceptibility testing is done using molecular methods these could give results quicker”*. *(IDI-2014)*.

*Another quote illustrates the problems of current diagnostic tools taking too long; “But another challenge is the delay in results*, *but I think the patients and the clinicians are not getting the right message*. *They think culture and drug susceptibility testing would only be a day’s work*, *which is not right culture on its own takes up to eight weeks”* **(***IDI*, *FGD-2014)*.

One male coordinator said that the process of centralization of supplies and commodities may be the cause of result delays.

*The following quote illustrates “Our programme is vertical*, *so when there is a shortage of slides we grieve*, *they should integrate so that some of these things could also be budgeted for by the councils at the district level*.*” (IDI-2014)*”

Some male and female participants said that, Central TB Reference Laboratory staff are overwhelmed.

*The following quote illustrates*; *“Central TB Reference Laboratory staff have been trying to perform their work but they are overloaded with many specimens from each side of the country*. *One reference laboratory for culture and drug susceptibility testing in Tanzania*, *I would suggest that*, *to identify another branch to reduce congestions which causes results delays” (FGD*, *2014)*.

### Communication between the Central TB Reference Laboratory and the districts

Communication between the Central TB Reference Laboratory and the district was mentioned to be very important to ensure people know what is going on. There is a need to improve communication between Central TB Reference Laboratory and peripheries.

*The following quote illustrates*; *“My perception the networking is not that strong*, *one reference laboratory for the whole country without internet and telephone services for sharing information*.*”(IDI-2014)*.

In addition, it was commented that result dissemination methods were not ideal as results are first sent to the Regional TB Coordinator and not the original requestors who manage the patients.

**Fig 2:**
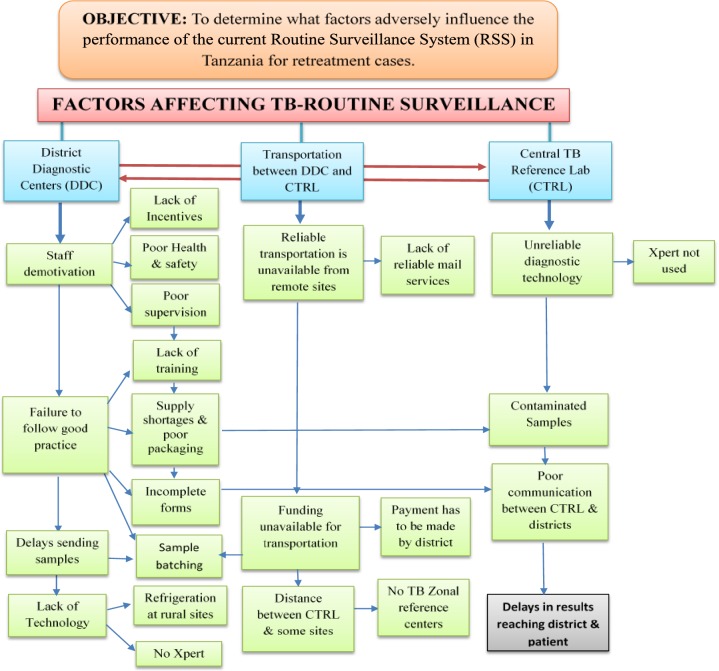
Key themes and sub-themes. Key: CTRL–Central TB Reference Laboratory. RSS–Routine Surveillance system.

The results from the qualitative research were reviewed and are presented according to the key themes in Fig 2. The three main health system areas were the District Diagnostic Centres, transportation between the District Diagnostic Centres and the Central TB Reference Laboratory, and the Central TB Reference Laboratory itself. In each of these areas key themes from the IDI’s and FGD’s were identified and then sometimes further divided into subthemes. Key themes identified at the District Diagnostic Centres were staff demotivation, failure to follow good practice, delays sending samples, and lack of appropriate technology. In relation to transportation between the districts and the Central TB Reference Laboratory the key themes were the unavailability of reliable transportation, funding unavailable for transportation, and the distance between the districts and the Central TB Reference Laboratory. At the Central TB Reference Laboratory, the key themes were unreliable diagnostic technology, contaminated samples, and poor communication with districts. These key themes and related sub-themes are shown in Fig 2 along with how many of these themes are interconnected.

## Discussion

The qualitative part of the study involved FGD’s and IDI’s with key stakeholders in every area involved in the routine surveillance system. It was designed to try and understand why some of the shortfalls in performance of the system observed in the quantitative analysis were occurring, and potentially to identify initiatives to address these weaknesses. The study results showed an association between the quantitative and qualitative results in many areas. For example, in the quantitative results of this study, it was found that the target of 100% of previously treated TB cases specimens being routinely investigated for drug resistant at the Central TB Reference Laboratory was far from being met. Only 61% of cases were sent for testing even in the best year of the three-year period investigated. This implies cases of drug resistance are likely to have been missed and full surveillance of the TB programme was not undertaken. This was also noted in the qualitative findings which showed the system of TB specimen transportation in Tanzania was a major problem in remote health facilities as there were no postal services and no reliable and frequent means of transport. In addition, this led to specimen batching at the district level hence delays in sending samples and diagnosis. This was associated with lack of funds to transfer specimens timely. This was also seen in the quantitative part of the study which showed the median transit time from sputum collection to arrival at the Central TB Reference Laboratory was over 7 days which may also have affected specimen culture viability. The proportion of samples processed within the recommended turnaround time at the Central TB Reference Laboratory for microscopy was 82.4%, for culture 42.4% and for drug susceptibility testing just 2.8%. This implies that more than 50% of the culture processed specimens and more than 90% of the drug susceptibility testing processed specimens were unable to get results in a timely. The consequence of this is a delay for patients getting onto appropriate treatment which in turn is likely to affect patient outcomes. This also correlates with the qualitative part where results delay was one of the aspects raised in the majority of interviews with health care workers. The current RSS was seen as poor in relation to the feedback and communication of the drug susceptibility testing results from the Central TB Reference Laboratory to the peripheries.

The overall turnaround time of drug susceptibility testing results is influenced by many aspects which include the laboratory procedures, but in Tanzania are also impacted by there being only one drug susceptibility testing laboratory for the whole country. Park et al [20] comment that the centralization of drug sensitivity testing has led to overwhelming workload at the Central TB Reference Laboratory, significantly contributing to ineffectiveness of the system in the country. Our study is in line with Harries et al who revealed that, problems often exist at all stages of the system from specimen transportation, processing and documentation.[21]. This study also showed the policy of all positive culture slopes to undergo drug susceptibility testing was adhered to in less than 50% of occasions, meaning drug resistant could be missed Our study is also consistent with Royce et al who noted that, delays due to many “handoffs” of specimens through different levels of health system and unintended consequences of health worker incentive payments can mean even if specimens reach the laboratory they may not be of adequate quality.[22].

The observed culture contamination rate in our study was 3.6% which was within the recommended level (0–5%) [3]. However, the Supra National & GLI Mission Visit Report in 2013 showed higher contamination rates (See attached report annex 1). The reason for this difference was mainly because of the way the contamination rate was calculated in our study which only considered contaminated if both slopes were contaminated instead of counting each contaminated slope. This suggests that the laboratory is probably missing the actual target rate and that contamination is still a concern.

The observed MDR TB rate of previously treated cases that did receive drug sensitivity testing was surprisingly high (72 out of 373 i.e. 19.3%—see Table 3). WHO estimates for Tanzania in 2016 were 4.7% of previously treated cases. The reason for this difference is unknown, but there are three possible explanations. Firstly, that the WHO estimate is an underestimate which is possible as it is only an estimate and is not based on a recent Drug Resistance Survey (note: one is currently underway). Secondly, that the observed figure in this study is an overestimate, potentially because those cases actually tested are not a random sample of all previously treated cases. They are potentially the ones that districts are most concerned about and actually have a higher probability of MDR-TB. The third possible explanation is that samples could have been contaminated due to the poor functioning of the RSS. Obviously it could be a combination of all three.

Addition qualitative observations from our study were comments on poor communication between the Central TB Reference Laboratory, weak supervision that contributed to low staff demotivation, request forms not being checked prior to specimen transportation and a general failure to follow good practices. These and the other key themes have been mapped in Fig 2.

The study was not without limitations; *firstly*, the routine data captured electronically was incomplete due to poor form filling that led to some difficulties in analysing information. It can be seen that the number of previously treated specimens recorded increased over the three years of our analysis, but the number received even in the peak was still low compared to the notified cases. There was a sharp decrease in the number of specimens received in 2011 and this was due to poor data quality. *Secondly*, in the qualitative study many of the experienced laboratory staff were not available for key informant interviews as a result of high staff turnover. The initial sampling of personnel for FGDs assumed achieving appropriate number of participants. [19]. In most of the selected districts the number were less than recommended resulting in exclusion of some sites. Time for the key informant interviews was limited due to their tight schedules and therefore some interviews were unavoidably hurried.

The findings of this study could assist the NTLP in its operational plans and funding allocations. Potential interventions that could be considered are to establish national guidelines for specimen packaging and referral arrangements to counteract contamination effects; increased awareness among district coordinators on the importance of sending specimens within the recommended period, decentralization of culture and drug susceptibility testing services and roll out more appropriate rapid TB laboratory techniques such as Xpert MTB/RIF. [23].

## Conclusions

The study findings provide a basis for identifying potential methods of improving the routine surveillance system and thereby providing more timely and appropriate management particularly for patients with MDR-TB. Based on these findings there is a need to address the identified shortfalls of the current system. The next step would be to pilot a revised a routine surveillance system that addresses some of these constraints and takes on board the use of Xpert MTB/RIF at some district diagnostic centres and the Central TB reference Laboratory with the focus on providing complete and timely information for management of previously treated TB patients.
